# A Comprehensive Review of Genistein’s Effects in Preclinical Models of Cervical Cancer

**DOI:** 10.3390/cancers16010035

**Published:** 2023-12-20

**Authors:** Matteo Nadile, Amanda Kornel, Newman Siu Kwan Sze, Evangelia Tsiani

**Affiliations:** 1 Department of Health Sciences, Faculty of Applied Health Sciences, Brock University, St. Catharines, ON L2S 3A1, Canada; 2 Centre for Bone and Muscle Health, Applied Health Sciences, Brock University, St. Catharines, ON L2S 3A1, Canada

**Keywords:** cervical cancer, genistein, proliferation, survival, apoptosis, signaling cascades

## Abstract

**Simple Summary:**

Cervical cancer is the fourth most commonly diagnosed cancer among women worldwide. While treatments exist to stop growth of cervical cancer in humans, they are often associated with major side effects and the development of resistance to therapy. Traditionally, plant-derived compounds have been used to treat many ailments, including cancer. The search for novel plant-derived chemicals is important, as they can potentially provide effective treatment with less severe side effects and importantly overcome drug resistance. Genistein and its analogues have been shown to decrease survival and proliferation as well as induce cell death in cell culture models of cervical cancer and reduce tumor volume in a mouse model. More pharmacokinetic studies and studies utilizing preclinical animal models of cervical cancer are required to increase our knowledge of genistein’s biological effects, prior to moving to human studies.

**Abstract:**

Cervical cancer is associated with persistent Human Papilloma Virus (HPV) infections and is the fourth most common cancer in women worldwide. Current treatment options; surgery, chemotherapy, and radiation, are often associated with severe side effects including possible infertility. Novel treatment options are required to help combat this disease and reduce side effects. Many plant-derived chemicals, including paclitaxel and docetaxel, are already in use as treatments for various cancers. Genistein is a polyphenolic isoflavone found in foods including soybeans and legumes, and studies have shown that it has various biological effects and anti-cancer properties. This review aims to summarize the existing studies examining the effects of genistein on cervical cancer. All relevant in vitro and in vivo studies are summarized, and the key findings are highlighted in the associated tables. Based on the available in vitro/cell culture studies reported here, genistein inhibits cervical cancer cell proliferation and induces apoptosis. Use of genistein in combination with radiation or chemotherapy agents resulted in enhanced response indicating radio- and chemo-sensitization properties. More animal studies are required to examine the effectiveness of genistein in vivo. Such studies will form the basis for future human studies exploring the potential of genistein to be used in the treatment of cervical cancer.

## 1. Introduction

### 1.1. Cervical Cancer

Cervical cancer is the fourth leading malignancy in women worldwide, with nearly 600,000 new cases diagnosed each year [1]. In Canada, it is the fourth most frequently diagnosed cancer among women aged 15 to 44 years and five-year survival rates are approximately 74% [2]. Globally, cervical cancer incidence and mortality rates are highest in low- and middle-income countries [1]. The predominant cause of developing cancer of the cervix is having persistent Human Papilloma Virus (HPV) infections, and HPV-16 and -18 have been identified as the most carcinogenic subtypes of the virus, accounting for 60% of cervical cancer cases. Cervical cancer can occur in HPV-negative patients; however, it is rare, and is often associated with prolonged oral contraceptive use [3]. HPV vaccines are the first line of defense in prevention of cervical cancer, and young women are encouraged to get annual pelvic exams and Pap smears (Papanicolaou test) every three years as early detection increases survival. Current treatment guidelines include surgery, radiation, and chemotherapy, with consideration taken for fertility sparing or nonfertility sparing methods. Chemotherapy agents currently in use include cisplatin, paclitaxel, docetaxel, topotecan, 5-fluorouracil, and carboplatin [3,4,5,6]. Paclitaxel, docetaxel, and 5-fluorouracil are drugs derived from natural sources such as the bark of the Pacific and European yew tree (*Taxus brevifolia* and *Taxus baccata*, respectively) and the marine sponge *Phakellia fusca* [7,8,9]. While these drugs are good examples of naturally derived treatments, they often result in chemoresistance, lowering their efficacy [10,11,12]. Additionally, other chemotherapy agents have major side effects, including loss of appetite, nausea, vomiting, mouth sores, hair loss, and fatigue [13]. Therefore, new treatment options are needed for cervical cancer patients.

### 1.2. Genistein

**Figure 1:**
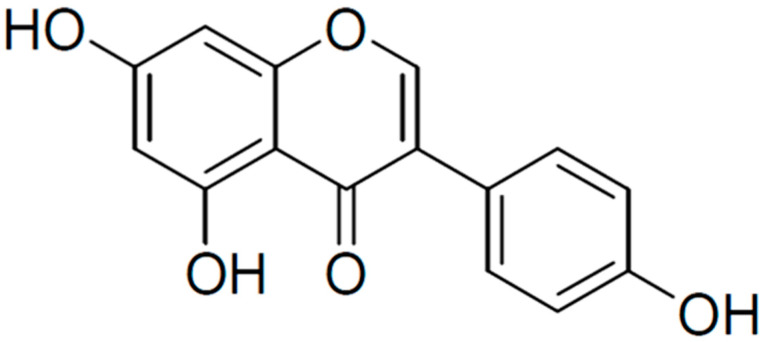
Chemical structure of genistein. Created in BioRender.com.

Genistein is a polyphenolic isoflavone (4′,5,7-trihydroxyisoflavone) present in soybeans, with an average concentration of 81 mg of genistein/100 g mature soybeans [14,15]. Legumes, such as chickpeas (garbanzo beans) and broad beans, are considered the second most important source and contain approximately 0.2–0.6 mg of genistein/100 g. Other fruits, nuts, seeds, and vegetables contain trace amounts (0.03–0.2 mg/100 g). Genistein has a diphenol structure (Figure 1) with the molecular formula C_15_H_10_O_5_ and resembles human endogenous estrogen [14,16]. There is evidence that genistein has antioxidant [17], anti-inflammatory [18], antibacterial [19], antiviral [20,21], antidiabetic [22,23], neuroprotective [24,25], and anti-cancer [26,27,28] effects. Additionally, in male mice, genistein increased sperm motility and number, suggesting it has beneficial effects in the reproductive system [29].

In this review, we aim to summarize the effects of genistein on cervical cancer. A search of PubMed was performed using the key words “genistein”, “cervical cancer”, or various combinations. Primary research articles were selected and are reviewed below and summarized in Tables.

## 2. Genistein against Cervical Cancer

### 2.1. Genistein against Cervical Cancer: In Vitro Studies

**Figure 2:**
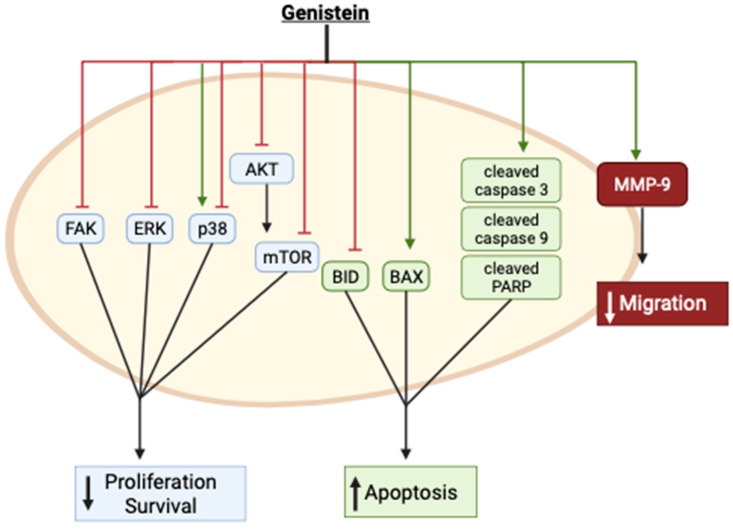
Summary of the effects of genistein in cervical cancer cells in vitro. The image was created using BioRender.com based on data from published studies [31,32,33,36,37,38,39,40].

Cervical cancer cell lines have been used to examine the effects of genistein using in vitro methods. These studies are summarized here, and the key findings are highlighted below in Table 1 and Figure 2.

HeLa and ME-180 cervical cancer cells treated with genistein had reduced cell growth and invasion, and increased cell cycle arrest and apoptosis [30]. The half-maximal inhibitory concentration (IC_50_) value for HeLa cells was 35 µM, with the majority of cells arrested in the S phase while for ME-180 cells, the IC_50_ value was 60 µM, with cells arrested in the G2/M phase (Table 1) [30].

Genistein treatment decreased colony formation in ME-180 (lethal dose (LD_50_): 11 µM) and CaSki (LD_50_: 24µM) cervical cancer cell lines. Cytochrome c was increased in both cell lines following treatment with 2.5 µM and 10 µM genistein, suggesting activation of apoptotic pathways [31].

Cervical cancer cells (HeLa) treated with genistein showed a decrease in cell survival (a 50% decrease was seen with 18.47 µM) and increased cell cycle arrest (increased the percentage of cells in S and G2/M phase) and apoptosis [32]. Western blot analysis revealed increased levels of cleaved poly (ADP-ribose) polymerase (PARP), a marker of apoptosis, after treatment with genistein in HeLa cells, while no cleaved PARP was seen in L929 cells (normal mouse fibroblasts). Treatment with genistein led to decreased protein expression of cyclin B1 and cyclin-dependent kinase 1 (CDK1), and decreased tyrosine phosphorylation of cell division control protein 2 (cdc2) (Table 1) [32].

Treatment of HeLa and CaSki cervical cancer cells with 5–80 µM of genistein for 24–48 h resulted in a significant inhibition of growth [33]. Treatment with genistein resulted in a time-dependent decrease in phosphorylated Akt and extracellular signal-regulated kinase (ERK), while phosphorylated p38 and c-Jun N-terminal kinase (JNK) were increased [33]. The authors used small molecule inhibitors in combination with genistein to view the effects on cell viability. Cell viability was significantly decreased (compared to cells treated with genistein alone) when both cervical cancer cell lines were treated with genistein and PD98059 (ERK inhibitor), while cell viability was increased (compared to cells treated with genistein alone) when SP600125 (p38 inhibitor) and genistein were used. These results suggest that genistein inhibits cell growth and decreases cell viability through decreasing the phosphorylation/activation of ERK, while increasing phosphorylation/activation of p38 and JNK [33].

Treatment of HeLa, CaSki, and C33A cervical cancer cells with 5–60 µM of genistein for 48 h resulted in inhibition of cell growth and decreased cell viability in all three cell lines, with the greatest response seen in HeLa cells [34]. Following treatment with genistein, the percentage of apoptotic cells (measured by flow cytometry) was increased in a dose-dependent manner, while the expression of pro-caspase-3, -8, and -9 was decreased. In line with these findings, the levels of cleaved PARP and the proapoptotic protein Bid (total) decreased while Bax levels were increased. Levels of Bcl2 remained unchanged (Table 1) [34].

Squamous cervical cancer cells (SiHa) treated with genistein showed reduced viability (IC_50_ value of 80 µM) [35]. Ethidium bromide staining revealed the formation of apoptotic bodies in these cells following genistein treatment, and internucleosomal DNA fragmentation was observed, consistent with apoptosis induction. Tumor-suppressor genes can be silenced by methylation; genistein (20 µM) was able to reverse *RARβ2* tumor suppressor gene methylation after 72 h and continued demethylation for six days. Genistein treatment also increased mRNA expression of *RARβ2* in SiHa cells [35]. This study provides evidence for genistein to not only act as an anti-cancer agent through the induction of apoptosis but also through its ability to de-methylate the tumor suppressor *RARβ2* leading to its re-activation. This is an important finding, as re-activation of tumor suppressors is a possible target for cancer therapies.

Genistein treatment decreased viability of HeLa cells and caused nuclear morphological changes characteristic of apoptosis: nuclear condensation, fragmentation, blebbing, and the appearance of apoptotic bodies [36]. Flow cytometry showed an increased number of cells in the G2/M phase following genistein treatment, suggesting cell cycle arrest at this stage. Treatment with genistein inhibited HeLa cell migration (wound closure assay). These results were correlated with a decrease in mRNA expression of MMP-9 and an increase in TIMP-1 expression [36].

HeLa cells treated with genistein resulted in decreased cell viability an effect that was associated with decreased expression of nuclear factor kappa B (NF-kB) p65 subunit [37]. Furthermore, phosphorylated levels of Akt, mammalian target of rapamycin (mTOR), p70S6K1, and Eukaryotic translation initiation factor 4E-binding protein 1 (4E-BP1) were significantly reduced with genistein treatment. These data suggest that genistein is able to decrease the rate of cell growth and proliferation, while potentially inducing apoptosis (Table 1) [37]. While this study indicates genistein’s ability to downregulate cell growth, and proliferation through inhibition of Akt- mTOR signaling, it fails to evaluate its ability to induce apoptosis or autophagy by examining caspases, PARP cleavage, and LC-3.

Cervical cancer cells (HeLa) treated with varying doses of genistein (0–100 µM) for 48 h were seen to have reduced cell viability and induction of apoptosis [38] as evidenced by the increased levels of cleaved caspase-3 and cleaved PARP. Furthermore, endoplasmic reticulum (ER) stress was shown by the increase in protein levels of GRP78, a molecular marker for ER stress, and C/EBP homologous protein (CHOP), a transcription factor [38]. These data suggest that genistein has the ability to induce apoptosis by increasing ER stress in HeLa cervical cancer cells.

Genistein (5 µM) decreased the viability of HeLa cervical cancer cells, an effect that was associated with increased expression of cleaved caspases -9 and -3, reduced mitochondrial membrane potential, and DNA fragmentation [39].

Treatment of HeLa cells with genistein resulted in inhibition of proliferation and migration that was associated with reduced levels of phosphorylated protein focal adhesion kinase (FAK) and paxillin, which are both involved in migration and invasion [40]. Moreover, β-catenin and vimentin levels, proteins involved in migration and metastasis, were reduced. Phosphorylation/activation of p38 and p42/44 mitogen activated protein kinase (MAPK) were also reduced with genistein treatment. mRNA expressions of FAK, paxillin, Snail, and twist were reduced with genistein treatment, suggesting suppression of the epithelial to mesenchymal transition (EMT) [40]. In another study, HeLa and CaSki cervical cancer cells treated with genistein had significantly reduced cell viability [41].

Sundaram et al. (2020) treated HeLa cervical cancer cells with 50 µM of genistein for 24 and 48 h and found increased cell cycle arrest, nuclear condensation, fragmentation, and the formation of apoptotic bodies [42]. Flow cytometry revealed an accumulation of cells in the G0 phase and cell cycle arrest in the G2/M phase in a dose-dependent manner. Nitric oxide (NO) levels were seen to increase significantly (2.37 µM) in cells treated with genistein. Genistein treatment upregulated many pro-oxidants while downregulating antioxidants at the transcript level (Table 1) [42].

HeLa cells treated with genistein (12.5, 25, 50, and 100 µM) had reduced proliferation and colony formation [43]. In addition, treatment with genistein caused a significant decrease in cell adhesion and migration/invasion (assessed with a wound-healing and a trans-well assay). RNA sequencing was performed to elucidate the mechanisms involved in these anti-cancer effects of genistein. Differentially expressed genes (DEGs) that were downregulated were associated with ribosomal subunits, metabolic processes, and mitochondrial translation. Western blot analysis showed a significant decrease in phosphorylated FAK and paxillin and total β-catenin, and vimentin protein levels with genistein treatment. A decrease in mRNA levels of FAK, PAXILIN, Snail, and TWIST was also seen with genistein treatment [43]. Based on these data, the authors concluded that genistein has anti-proliferative and anti-metastatic properties against cervical cancer through inhibition of the FAK/paxillin pathway.

While all the above studies provide evidence that genistein inhibits proliferation and survival of cervical cancer cells in vitro, one study suggests it may promote the growth of cervical cancer cells.

Chen et al. (2018) found that HeLa cervical cancer cells treated with low concentrations of genistein (0.001–1 µM) showed an increase in cell proliferation [44]. Genistein altered the cell cycle by decreasing the portion of cells in G1 phase while increasing the portion of cells in the S phase. The apoptotic rate was significantly lower in genistein-treated cells. Western blot analysis revealed an increase in estrogen receptor alpha ERα, phosphorylated Akt, and nuclear NF-kB p65 protein levels with 0.1 µM genistein treatment [44]. The effects of genistein were attenuated in the presence of an inhibitor of ERα (MPP), a phosphoinositide 3 kinase (PI3K)-Akt inhibitor (LY294002), or a nuclear NF-kB p65 inhibitor (PDTC), indicating that the increased cell viability seen with genistein is due to the activation of ERα-PI3K/Akt -NF-kB p65 signaling cascade.

**Table 1:** Effects of genistein against cervical cancer: in vitro studies summarized.

Cell Line	Genistein Concentration/Duration	Effect	Reference
HeLa ME-180	IC_50_ 35 and 60 µM	↑ Cell cycle arrest S phase (HeLa) ↑ Cell cycle arrest G2/M phase (ME-180) ↓ Invasion ↑ Apoptosis	[30]
ME-180 CaSki	10–40 µM 6 days (colony formation) 48 h	↓ Colony formation ↑ Cytochrome c	[31]
HeLa	25–150 µM 48 h	↓ Cell survival ↑ Cell cycle arrest ↑ PARP cleavage ↓ Cyclin B1 ↓ cdc2 ↓ P-Tyr cdc2	[32]
HeLa CaSki	5–80 µM 24–48 h	↓ Cell growth ↓ Cell viability ↓ p-Akt ↓ p-ERK ↑ p-p38 ↑ JNK	[33]
HeLa CaSki C33A	5–60 µM 24–48 h	↓ Cell viability ↑ Apoptosis ↓ Procaspase-3, -8, -9 ↑ Cleaved PARP ↓ Bid ↑ Bax	[34]
SiHa	80 µM 48–72 h	↓ Cell viability ↑ Apoptosis ↓ *RARβ2* methylation	[35]
HeLa	75, 100, and 150 µM 24 and 48 h	↓ Cell viability ↑ Apoptosis G2/M Cell cycle arrest ↓ Migration ↓ MMP-9 mRNA expression ↑ TIMP-1 mRNA expression	[36]
HeLa	25 µM 24 h	↓ Cell viability ↓ NF-kB p65B ↓ p-mTOR ↓ p-p70S6K1 ↓ p-4E-BP1 ↓ p-Akt	[37]
HeLa	0–100 µM 48 h	↓ Cell viability ↑ Apoptosis ↑ Cleaved caspase-3 ↑ Cleaved PARP ↑ GRP78 ↑ CHOP	[38]
HeLa	5 µM 24–72 h	↓ Cell viability ↑ Cleaved caspase -9 ↑ Cleaved caspase -3 ↑ DNA fragmentation	[39]
HeLa	0–150 µM 24–48 h	↓ Proliferation ↓ Migration ↓ Invasion ↓ p-FAK ↓ p-paxillin ↓ p-p38 ↓ p-p42/44 ↓ β-catenin ↓ Vimentin ↓ p-paxillin mRNA levels ↓ p-p38 mRNA levels ↓ Snail mRNA levels ↓ twist mRNA levels	[40]
HeLa CaSki	120–180 µM 24 h	↓ Cell Viability	[41]
HeLa	50 µM 48 h	↑ Cell cycle arrest G2/M phase ↑ Nuclear condensation ↑ Nuclear fragmentation ↑ NO levels	[42]
HeLa	0.01–1 µM 48–72 h	↑ Cell proliferation ↑ S-phase arrest ↓ Apoptosis ↑ p-Akt ↑ Erα ↑ Nuclear NF-kB	[44]

Table legend: ↑ increased, ↓ reduced, p- phosphorylated.

Collectively, these in vitro studies provide evidence of inhibition of cervical cancer cell proliferation and survival and induction of apoptosis with genistein treatment. These effects were associated with inhibition of FAK, ERK, Akt, mTOR, and BID while the apoptotic markers Bax and cleaved caspases and PARP were enhanced (Figure 2). p38 was found to be activated by genistein in one study [33] and inhibited in another [40]. The reasons for these differences are not clear, as both studies examined the effects in the same cervical cancer cells (HeLa). Furthermore, genistein inhibited cervical cancer cell migration, an effect that was correlated with enhanced MMP-9 protein levels (Figure 2).

### 2.2. Genistein Analogues and Nanoparticles against Cervical Cancer: In Vitro Studies

Genistein has low solubility and bioavailability. In an effort to increase bioavailability and drug uptake, which will increase genistein’s effectiveness as an anti-cancer drug, some researchers have examined genistein analogs or nanoparticle delivery options.

Nanoparticle technology has been employed for the encapsulation and delivery of compounds with low absorption and bioavailability. Genistein-loaded nanoparticles using inulin-stearic acid have shown potent and specific toxicity against human colorectal cancer cells [45]. In addition, genistein encapsulated in zein/chicory polysaccharide nanoparticles had a greater inhibition of hepatic (HepG2) cell proliferation when compared to parent genistein [46]. Studies which examined genistein analogues and conjugates against cervical cancer are presented and summarized below and in Table 2.

Xiong et al. (2015) synthesized seven analogues of genistein and examined their effects in human cervical cancer (HeLa) cells. Each of the analogues had a much lower inhibitory effect on HeLa cell proliferation than the parent compound. The cell growth inhibition ranged from 3.4 to 13.1% when exposed to 1 µM of the analogues and 8.5 to 26.8% with 10 µM, as compared to the parent compound genistein which showed a cell growth inhibition of 9.9% (1 µM) and 54.0% (10 µM) (Table 2) [47]. Genistein’s effect on proliferation had IC_50_ value of 10.0 ± 1.5 µM. The IC_50_ values of the different analogues were in the range of 51.3 µM ± 5.0 to 1000 µM. These high IC_50_ values clearly suggest the analogues are much less effective. The data of this study indicate that the parent compound, which contains hydroxyl groups located at carbon-5 and carbon-7, is more effective, indicating an important role of these hydroxyl groups on its antiproliferative activity [47].

Genistein was encapsulated in folic-acid-conjugated chitosan nanoparticles (FGCN) with a hydrodynamic diameter less than 200 nm, suggesting they are small enough to penetrate tumor tissues and resist rapid clearance. Cervical cancer cells, HeLa, had significantly decreased cell viability when treated for 24 h with FGCN compared to cells treated with free genistein (IC_50_ 14.6 µg/mL and 33.8 µg/mL, respectively) [48]. Cell number was decreased following treatment with FGCN, and visual examination revealed rounded cells suggesting damage and/or cell stress. Flow cytometry analysis showed a significant increase in the percentage of apoptotic cells following treatment with FGCN compared to free genistein. Fluorescence analysis showed a significantly higher cellular uptake of conjugated genistein over four hours [48]. Based on these findings, the authors concluded the folic acid-conjugated chitosan nanoparticles increased the toxicity of genistein to the cervical cancer cells. Folic acid receptors are present in high numbers on cervical cancer cells, so conjugating the nanoparticles to folic acid increases the affinity to folic acid receptors, thereby increasing drug uptake to the cells and increasing the biological effects. This paper provides evidence for the benefits of conjugating natural compounds to molecules with known cell membrane receptors to increase their effectiveness as cancer treatments.

**Table 2:** Effects of genistein analogues and nanoparticles against HeLa cervical cancer studies summarized.

Cell Line	Nanoparticle/Conjugate	Effect	Reference
HeLa	1 & 10 µM 3 days	↓ Proliferation ↑ Inhibitory rate	[47]
HeLa	Folic acid chitosan conjugate	↓ Cell viability ↑ Apoptosis	[48]

Table legend: ↑ increased, ↓ reduced.

### 2.3. Genistein in Combination with Other Compounds

Human cervical cancer cells (HeLa) co-treated with genistein (5 µM) and resveratrol (5 µM) had reduced percentage of viable cells when compared to either compound alone (genistein 5 µM, resveratrol 25 µM) (Table 3) [39]. This reduction in viability was associated with increased cleavage of caspases -9 and -3. Genistein-induced apoptosis was significantly enhanced when resveratrol was co-applied. With the co-treatment of genistein and resveratrol, there was a significant decrease in gene expression of HDM2, a negative regulator of p53 [39]. These results provide evidence that genistein is able to act synergistically with resveratrol to enhance the effects of either compound alone.

Treatment of cervical cancer cells (HeLa and CaSki) with the chemotherapy agent cisplatin in combination with genistein resulted in a significant decrease in cell viability compared to either agent alone [41]. Cells co-treated with cisplatin and genistein had decreased protein levels of phosphorylated ERK1/2 and total Bcl2, as well as increased levels of p53 and cleaved caspase 3. These results suggest genistein is able to enhance cisplatin treatment by inducing apoptosis through inhibition of ERK1/2 phosphorylation/activation and increased p53 [41].

**Table 3:** Summary of the effects of genistein in combination with other compounds.

Cell Line	Genistein/Combination	Effect	Reference
HeLa	Genistein 5 µM 24–72 h Resveratrol 5 µM	↓ Cell viability ↑ Cleaved caspase-9 ↑ Cleaved caspase-3 ↓ Mitochondrial membrane potential ↑ DNA fragmentation ↓ HDM2 gene	[39]
HeLa CaSki	Genistein 80 µM 24 h Cisplatin 6 µM	↓ Cell viability ↓ p-ERK1/2 ↑ p53 ↑ Cleaved casp-3 ↓ Bcl2	[41]

Table legend: ↑ increased, ↓ reduced, p- phosphorylated.

### 2.4. Genistein in Combination with Radiotherapy

Human cervical cancer cells (ME-180 and CaSki) had increased sensitivity to radiotherapy when pre-exposed to genistein; ME-180 cells showed the greatest sensitivity [31]. Genistein treatment for 48 h resulted in G2M cell cycle arrest, which may be involved in its radio-sensitization effect. Western blot analysis showed that genistein (10 and 40 µM) combined with radiation (500 cGy) led to decreased protein expression of Mcl-1 and total and phosphorylated/activated Akt (Ser473 and Thr308) (Table 4) [31].

Combined treatment of HeLa cells with genistein and 400 cGy radiation (IR) resulted in decreased growth that was greater than with genistein or IR alone [49]. Flow cytometry analysis showed that the combined treatment resulted in significantly increased number of apoptotic cells compared to each approach alone. Combination treatment revealed a decrease in both mRNA and protein levels of survivin; and decreased protein levels of Cyclin-B. Together these results show that genistein is able to inhibit the growth of HeLa cervical cancer cells through cell cycle arrest and reduced survivin levels and this effect is enhanced when administered in conjunction with low dose radiation therapy [49]. This paper provides evidence that genistein has potential as a radiation sensitizer.

**Table 4:** Summary of the effects of genistein in combination with radiotherapy against cervical cancer cell lines.

Cell Line	Genistein/Combination	Effect	Reference
ME-180 CaSki	Genistein 10–40 µM 200, 500, or 800 cGy radiation	↑ Cytochrome c induction ↓ Mcl-1 protein expression ↓ Akt & p-Akt protein	[31]
HeLa	Genistein 20–100 µM 400 cGy radiation	↓ Cell viability ↑ Apoptosis ↓ Survivin mRNA and protein	[49]

Table legend: ↑ increased, ↓ reduced, p- phosphorylated

### 2.5. Genistein against Cervical Cancer: In Vivo Animal Studies

**Figure 3:**
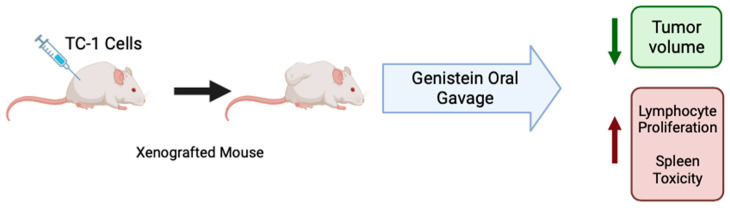
Summary of the effects of genistein in cervical cancer in animals. Figure created with BioRender.com based on data from Ghaemi et al. (2012) [50].

C57BL/6 mice xenografted with cervical cancer TC-1 cells, which are HPV-16 E7+, were treated with genistein (20 mg/kg) by oral gavage daily for 10 days before and 10 days after tumor cell injection [50]. Overall, the mice that received genistein had a significantly lower tumor volume after 10, 20, and 30 days of the study than the dimethyl sulfoxide (DMSO) control group. Mice that received genistein treatment increased lymphocyte proliferation as compared to mice which received water or DMSO (control and vehicle control). Spleen cells from treated mice had increased levels of cytokine IFN-y compared to controls, indicating that genistein induces a T helper cell type 1 profile (Table 5). Together, these results suggest that orally administered genistein has immunomodulatory effects against cervical cancer by increasing proliferation of splenic lymphocytes (Figure 3) [50].

**Table 5:** Effects of genistein against cervical cancer: in vivo animal studies.

Model	Genistein Concentration/Duration	Effect	Reference
TC-1 (cervical cancer cells) Injected subcutaneously in the right flank of mice	20 mg/kg Oral gavage Daily	↓ Tumor volume ↑ Lymphocyte proliferation ↑ cytotoxicity in spleen ↑ IFN-y concentration	[50]

Table legend: ↑ increased, ↓ reduced.

## 3. Discussion

### 3.1. General Overview

The in vitro studies summarized in this review provide evidence that the isoflavone genistein inhibits cervical cancer cell proliferation and survival and induces apoptosis. The concentrations and durations varied between studies and the range of concentration examined was 5–150 µM, with the most significant inhibitory effects occurring at concentrations ranging from 25–100 µM. The exact mechanism of action of genistein is not fully elucidated. The combined evidence from these studies suggests genistein acts to inhibit proliferation and survival of cervical cancer cells through inhibition of ERK, Akt and mTOR. Genistein treatment increased the number of apoptotic cells through increasing activity of caspases -3 and -9 and the subsequent cleavage of PARP. Treatment with genistein also decreased migration of cervical cancer cells by increasing MMP-9 activity.

### 3.2. Uncertainties and Variations

As mentioned above, varying concentrations of genistein were used in the studies presented herein. Genistein’s anticancer effects were mainly seen with concentrations ranging from 25–100 µM. These micromolar concentrations used beg the question of whether they are relevant to in vivo models. In one study, genistein was effective at concentrations as low as 5 µM [39]. In that combination study [39], the authors suggest that lower concentrations of genistein alone can elicit moderate cytotoxic effects on cervical cells, but they can work synergistically with other bioactives, such as resveratrol. Researchers are encouraged to examine lower concentrations of genistein alone as well as in combination with other plant-derived compounds but should be aware of off-target effects of each chemical. On the other hand, in one study, low concentrations of genistein were found to increase cervical cancer cell proliferation [44].These data suggest that cells exposed to low concentrations of genistein for short periods of time can induce pro-cancer effects. These effects can potentially be attributed to genistein’s estrogen-like properties [14]. Further studies at low concentrations of genistein, that are likely more relevant in vivo, should be conducted in order to discern the exact effects of genistein in cervical cancer.

Finally, genistein’s ability to act as a potential treatment for cervical cancer remains uncertain as there are limited data in vivo. After examining the literature, we were only able to find one in vivo study examining genistein’s effects on mice ectopically xenografted with TC-1 cervical cancer cells. More studies should be conducted to increase the confidence of genistein’s antitumor ability as well as testing the translatability of in vitro studies to in vivo models. Moreover, in order to mimic the tumor microenvironment, a more physiologically relevant in vivo model is required. Orthotopic xenografts of cervical cancer [51] would provide a more accurate model to study growth, metastasis, and cellular signaling pathways.

### 3.3. Bioavailability

Genistein, similar to other polyphenolic compounds, has low bioavailability [15]. Male FVB mice were given genistein (20 mg/kg) either through intravenous bolus injection or oral administration, and plasma genistein levels of 57.70 ± 21.84 µM (rapidly declined), and 0.71 ± 0.22 µM (at 75 min), respectively, were seen [52]. This study has shown that the majority (up to 80%) of genistein is converted to metabolites such as glucuronides and sulfates. Despite its low bioavailability, genistein has a long half-life of 46 h in vivo [52]. In limited human studies, genistein metabolites were measured in urine and found to be predominately glucuronides and sulfates. The unconjugated genistein levels in urine were very low [53,54,55]. Unfortunately, there are no human studies examining plasma levels of genistein after oral administration. The one study from mice [52] suggests that oral administration (20 mg/kg) of genistein results in plasma levels of 0.71 µM.

Many studies provide evidence for genistein to act as an anti-cancer agent, including those discussed here which examined genistein’s effects in cervical cancer. However, there remains some evidence to suggest it may increase the rate of cancer growth. Genistein has been shown to increase growth of breast [56] and prostate cancer [57] cells. Genistein also increased tumor growth in mouse models of breast [56,58] and prostate [57] cancer. It is absolutely required to fully understand the effects and the molecular mechanisms by which genistein is acting in cells before it can be suggested as a cancer treatment option. It is important to examine the effects of different concentrations of genistein and especially the effects of low concentration in the range of 0.1–1 µM, as low concentration may have pro-cancer effects, while higher concentrations of 5–150 µM have anti-cancer effects. Similarly, more animal cervical cancer xenograft studies are required to examine the effects and mechanisms of genistein in vivo.

### 3.4. Organoids

Cell cultures are the most commonly used tool for studying diseases such as cancer in vitro. This technique allows examination of a drug’s effect on a specific cell type and can provide a lot of important pre-clinical information. However, many cancer cell lines which are commercially available were established decades ago and may have lost their integrity, therefore their value is limited. Stem-cell-derived and patient-derived cell cultures may provide a better, more accurate model for examining drug effects on cervical cancer. These models, however, are limited in that they can only be maintained for short time periods (2–3 weeks). Pre-clinical models are an important first step in understanding diseases and research into establishing better models is ongoing. Organoid cultures can be established from ecto- and endocervical malignant tissues, grown as 3D organoids, and grown in culture long term [59]. This model of organoids provides an effective tool for the study of cervical cancer [60]. Advances in 3D modeling and the ability to replicate the tumor microenvironment and architecture more closely with these models have made 3D organoid models the new gold standard in pre-clinical drug studies [61]. Organoid models can be used to study drug sensitivity as well as radiation sensitivity, and when derived from patient cells, can be used to create personalized treatment plans, leading to better outcomes [62,63].

## 4. Conclusions

The studies presented in this review provide evidence that genistein inhibits cervical cancer cell proliferation and induces their apoptosis. Genistein also has the potential to act as a radiation and chemotherapy sensitizer. Future studies should examine the effects of genistein in animal models of cervical cancer and its bioavailability and metabolism. More mechanistic studies are needed to fully elucidate the cell signaling and proteins involved in genistein’s actions. Animal studies utilizing cervical cancer cell xenografts are required to understand the effects of genistein in vivo. Additionally, clinical human studies must be performed before any recommendations could be made for its use in treating cervical cancer patients.
